# Development of a workforce self-assessment tool for public health emergency preparedness

**DOI:** 10.1093/eurpub/ckae030

**Published:** 2024-04-01

**Authors:** Jessica S Hayes, Marta Barreto, Laura Kalin-Mänttäri, Ricardo Mexia, Máire A Connolly, Liina Voutilainen

**Affiliations:** Global Health, School of Health Sciences, University of Galway, Galway, Ireland; Departamento de Epidemiologia, Instituto Nacional de Saúde Doutor Ricardo Jorge, Lisboa, Portugal; Department of Health Security, Finnish Institute for Health and Welfare, Helsinki, Finland; Departamento de Epidemiologia, Instituto Nacional de Saúde Doutor Ricardo Jorge, Lisboa, Portugal; Global Health, School of Health Sciences, University of Galway, Galway, Ireland; Department of Health Security, Finnish Institute for Health and Welfare, Helsinki, Finland

## Abstract

**Background:**

In collaboration with six European public health agencies as part of the PANDEM-2 consortium, we have developed and validated a self-assessment tool that captures the workforce capacities and capabilities needed at the institutional level within National Public Health Institutes (NPHIs) to deal with public health emergencies.

**Methods:**

The work carried out in this study included (i) a review of existing tools for workforce assessment, (ii) focus group discussions and interviews to map the experiences and needs of NPHI’s, (iii) the development of a tool for NPHI’s to assess their workforce capacity and capabilities in public health emergency preparedness (PHEP) and (iv) refinement of the assessment tool via a Delphi study.

**Results:**

Capacity markers were identified to assess the workforce required for PHEP functions and the availability of surge capacity during a public health emergency. The tool also enables NPHIs to analyze gaps in PHEP staff competencies. The assessment scores can assist NPHI pandemic preparedness by identifying and prioritizing training and recruitment needs.

**Conclusions:**

In line with EU Regulation 2022/2371 on serious cross-border threats to health, article 11 Training of healthcare staff and public health staff, Member States (MS) are tasked with assessing current workforce capacity and capability gaps. The PANDEM-2 workforce self-assessment tool aligns with this requirement and will support effective planning and development to strengthen the public health workforce capacity in EU MS.

## Introduction

The COVID-19 pandemic demonstrated the magnitude of the threat posed by communicable disease outbreaks and the demands on public health workforces to adequately respond to such outbreaks. Assessment of the public health workforce is a prerequisite for improving our ability to identify gaps, forecast future workforce needs, guide workforce development and policy and ultimately strengthen the workforce capacity. This is embedded within the International Health Regulations (IHR, 2005)^1^ Monitoring and Evaluation Framework which was developed to support countries in assessing capacity. The framework comprises the World Health Organization (WHO) States Parties Annual Reporting (SPAR)^2^ tool for mandatory annual reporting and includes the Joint External Evaluation (JEE),^3^ for assessment and testing of IHR core capacities. Moreover, the Health Emergency Preparedness Self-Assessment (HEPSA) tool^4^ was developed by the European Centre for Disease Prevention and Control (ECDC) to aid countries in improving their level of PHEP by evaluating levels of preparedness, identify potential gaps, identify vulnerabilities and detect areas for improvement at a national level.

Despite the availability of these tools, challenges remain. There have been several attempts to classify the public health workforce into groups, however, the broad scope of public health makes this challenging. Consequently, a standardized European classification is not available. This leads to a lack of reliable data in many countries and difficulties in obtaining information. Importantly, current health security assessment tools address national-level workforce capacity, whereas public health agencies require a tool applicable at the ‘institutional level’, allowing more granularity for assessment and planning. This is key as functional National Public Health Institutes (NPHIs) are critical for effective outbreak response, guiding evidence-based health policies and strategies, building health workforce capacity, implementation of the IHR (2005),^1^ and managing a robust disease surveillance system.^5–7^

The PANDEM-2 project (Pandemic Preparedness and Response, 883285) aims to enhance pandemic preparedness through innovations in information technology and training, which includes the development of a suite of tools for readiness assessment. We worked with six national European Union (EU) Member State (MS) NPHIs to develop and validate a tool to inventory the workforce capacity both in quantitative (full-time equivalents, FTE’s) and in qualitative (competency) terms. This self-assessment tool will enable European NPHIs to analyze deficits in public health emergency preparedness (PHEP) capabilities at the institutional level, ensuring effective planning to strengthen the workforce capacity in EU MS against future public health threats.

## Methods

The work carried out in this study was conducted in four stages: (i) a review of existing tools for workforce assessment, (ii) a focus group discussion and interviews to map the experiences and needs of NPHIs, (iii) development of a tool for NPHIs to assess their workforce capacity and capabilities in PHEP and (iv) refinement of the assessment tool through a Delphi study.

Firstly, current methodologies to assess how pre-existing tools enumerate, map and assess the workforce were reviewed. Three main sources were identified: (i) WHO, (ii) ECDC and (iii) other. These are explored in the results section.

Secondly, semi-structured interviews were conducted with participants from six EU MS (Finland, the Netherlands, Sweden, Romania, Germany and Portugal) NPHI to evaluate experiences and expectations on workforce assessment. Participants with working knowledge of one, or several of the following: (i) current practices to assess the PHEP workforce at the NPHI; (2) the use of tools by WHO, ECDC or other organizations that involve workforce assessment, such as the WHO JEE and/or IHR SPAR, ECDC HEPSA or the assessments of workforce capacity and training needs for the prevention and control of communicable diseases in the EU/EEA^8^; or, (iii) has an understanding of assessment needs of the NPHI at different PHEP domains (laboratory, statistics/modelling, communications, etc.) were targeted. Interviews were held online in December 2022. Three over-arching themes were explored: (i) participant experiences with pre-existing workforce methods, (ii) current NPHI best practices for workforce assessment and planning and (iii) specific needs of NPHI for a workforce assessment tool. Participants were informed of the anonymity of their answers so that no person or country could be identified from the report. A full list of interview questions can be found in the Supplementary information.

A pilot version of the workforce self-assessment tool was developed. The metrics for assessing the capacities and capabilities relevant to the PHEP context in NPHI were selected based on existing literature, the focus group discussion and interviews, as well as internal discussion within the working group. We selected capacity and capability indicators that are specifically relevant to the PHEP context in NPHIs. Consequently, the workforce indicators in the WHO SPAR and JEE tools, as well as the ECDC HEPSA tool were omitted from the PANDEM-2 tool as they were designed to describe country-level capacities and capabilities. To fulfill the aim of including cross-cutting capabilities in the tool, we reviewed other resources, such as the Council on Linkages between Academia and Public Health Practice: Core Competencies for Public Health Professionals (2014, 2021)^9^ and de Beaumont Foundation: Adapting and Aligning Public Health Strategic Skills (2021).^10^ As PHEP is often only one part of a professional’s tasks in an NPHI, we chose FTEs to enumerate workforce capacity. For assessing competencies, we chose to use a similar approach as the Hennessy–Hicks Training Needs Analysis (TNA),^11^ a widely used tool endorsed by the WHO.

Finally, the pilot tool was validated by participants from the six EU MS NPHI (*n* = 14) using a modified Delphi study design based on the RAND/UCLA appropriateness method.^12^ Ten of 14 respondents had been employed by the NPHI 10+ years, two for 5–10 years and two for <5 years and all had an active role in PHEP within their institute. Each indicator was scored using a 9-point Likert scale (1 = not relevant at all, 9 = highly relevant), or ‘I don’t know’. Agreement between respondents was calculated as the percentage of given scores in the highest tertile (7–9)/all given scores. Indicators with a median score >7 and agreement >70% were included in the final tool. Indicators with a median score >7 and agreement <70% required discussion and voting in a consensus round. Indicators with a median score ≤7 were excluded. In the consensus round, only indicators where >70% consensus was met were included in the final tool.

## Results

### Review of existing tools for workforce assessment

A total of eight tools related to workforce capacity and capability of NPHI’s were reviewed.

These were divided into three subcategories: (i) WHO, (ii) ECDC and (iii) other. A summary overview of each tool reviewed and key information can be found in the Supplementary information.

### Focus group findings

#### Experiences of NPHIs on existing assessment methods

At the time of the interview, only two of the participating countries (Finland and Germany) had undergone the WHO JEE. In one country (The Netherlands), a JEE had been postponed due to COVID-19. Two experts were involved in the development of the tool. One expert had contributed to three JEEs as an evaluator, mainly in low-economic status countries. Participants agreed that the JEE tool outputs on workforce capacity and capability are mainly qualitative. The JEE tool was seen as straightforward to use, but the required data are not always available.

Several participants had experience with SPAR. One participant found SPAR difficult to implement at NPHIs as it is based on national level indicators which are not easily translated to the institutional level. It was noted that the revision of SPAR last year improved the assessment, as indicators are now better defined to provide a more accurate reflection of workforce capacity and capabilities.

Views on the HEPSA tool were mixed: one participant organization suggested it provides more quantitative data regarding workforce capacity and capabilities, but another one found that the tool does not measure workforce capacity, but only equipment. One NPHI had been involved in the development of HEPSA, ranking indicators for the tool. The NPHI had organized in-house simulation training and found the HEPSA tool useful but concluded that personnel would require training on the tool prior to implementation.

With current assessment tools, the participants identified several gaps in the aftermath of COVID-19. Firstly, more capacity to analyze the available data was needed. Secondly, experience from different sectors, such as logistics and pharmacy would have been valuable regarding COVID-19 vaccine procurement and roll-out. Thirdly, the capacities and capabilities for communication were insufficient, and fourthly, legal expertise was needed to understand legislation with regard to pandemic plans. Conversely, it was noted that due to the dynamic and unpredictable nature of a pandemic, it is difficult to say with certainty, which indicators would be ‘future-proof’.

#### Current practices in workforce capacity and capability planning at NPHIs

Several participants had experience in workforce planning in their organization. It was agreed that general preparedness plans incorporate surge capacity for the workforce, but these aspects are generic and do not specifically apply to NPHIs. Many stated that the surge capacity required during the COVID-19 pandemic had been recruited on an ad hoc basis, without a pre-existing procedure. In two countries, staff had been seconded from local PH services to the NPHI, and in several institutes, personnel were ‘borrowed’ from other departments. One NPHI reported having a database of public health system personnel, from which they can request personnel to NPHI. However, this database is not PHEP-specific, and there is a shortage of personnel with epidemiological expertise. Some of the relocated staff had received previous training for competencies required in PHEP, and it was agreed that these competencies should be maintained for the future. Training was also provided on an ad hoc basis, as some staff members lacked the required skills.

Learning from the COVID-19 crisis, NPHIs are making plans to prepare for rapid upscaling in future crises. This includes implementing ‘reserve training’ during peacetime to provide a baseline level of knowledge to provide a pool of personnel that can be redeployed with minimal requirement of training and identifying suitable personnel from a PHEP-specific database. With regards to workforce funding, the NPHIs found it difficult to affect budget decisions on the ministry or strategic level by merely stating the number of persons needed. Instead, it was considered more effective to state which functions the PHEP personnel can perform with current funding, and which can be performed with increased funding.

#### Expectations of NPHIs for an ideal tool for workforce assessment

Based on the experiences from the COVID-19 crisis, a gap was clearly identified: NPHIs require tools for identifying and predicting workforce requirements, especially for PHE situations. The existing tools generally address national-level workforce capacity, whereas NPHIs require a practical tool applicable to the organization level, allowing more granularity for assessment and planning.

Using the tool should enable determining the right number of personnel that possess the needed competencies for PHEP functions, as well as identifying and addressing training needs. One participant suggested that the tool could couple capacity (absolute numbers) with competency, identifying personnel needed for specific roles and hours needed to complete the role, providing a better assessment of capability than the existing assessment tools. One participant suggested the tool to include a three-tiered system as in a national Emergency Operations Centre (Level 1: work in the usual core group; Level 2: draw resources from the same institute to upscale the staff; Level 3: recruit outside of the organization).

It was agreed that the PANDEM-2 tool should be customizable according to national guidelines and needs, fulfilling the mandates and roles of different agencies/countries. The tool should be adjustable over the years and from gained experience. Regarding the use of the tool, there was a consensus on a hybrid approach: the process could be started by a small team, including experts and decision-makers to ensure the outputs of the tool have practical and financial support. The human resources department was suggested as the initiation point, with technical support from the other units (e.g. finances). The work of this ‘core’ team could be followed by a consensus meeting held at the institutional/department level. Involvement of the Ministry of Health was also suggested.

Using the tool, three years was considered as a suitable time for repeating the assessment and planning, as an annual assessment would be too much of a burden and would not offer sufficient time for gaps to be addressed. It was suggested that the assessment could follow the same period as the upcoming EU country reporting on prevention, preparedness and response planning (based on Article 7 in EU Regulation 2022/2371 on serious cross-border threats to health).^13^

### Pilot tool development

**Figure 1:**
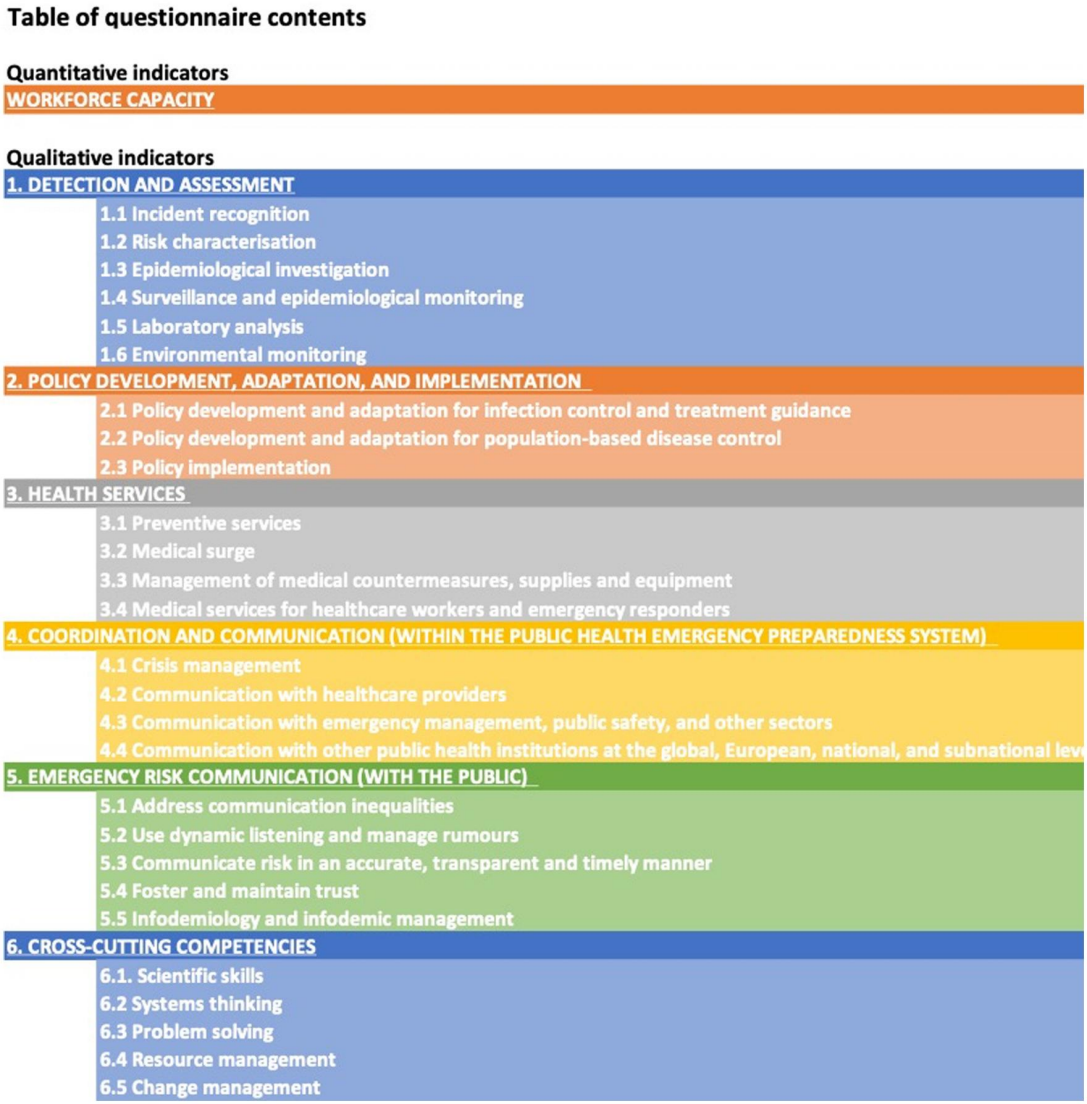
Overview of the quantitative and qualitative indicators included in the PANDEM-2 pilot workforce self-assessment tool. A full outline can be found in the Supplementary information

An overview of the indicators included in the pilot tool is outlined in figure 1. A detailed outline of the pilot tool can be found in the Supplementary information.

In total, 115 qualitative competency indicators specific to the PHEP context were selected for the pilot tool. This included 102 competencies from the Public Health Emergency Preparedness: Core competencies for EU MSs (2017)^14^ framework, divided under five categories: (i) detection and assessment, (ii) policy development, adaptation and implementation, (iii) healthcare services, (iv) coordination and communication (within the PHEP system) and (v) emergency risk communication (with the public). As the framework had been developed prior to the COVID-19 pandemic, we sought also indicators to reflect the requirements that emerged during the pandemic. Five such indicators were identified in the new domain ‘Infodemiology and infodemic management’ in Core competencies in applied infectious disease epidemiology in Europe,^15^ and were included under the category 5: Emergency risk communication (with the public) of the PANDEM-2 pilot tool.

Cross-cutting competencies that would be relevant in crisis situations were also included. Eight additional indicators in five subcategories were identified: (i) scientific skills, (ii) systems thinking, (iii) problem solving, (iv) resource management and (v) change management, from two sources: the Core Competencies for Public Health Professionals by the Council on Linkages Between Academia and Public Health Practice^9^ and the strategic skills comprised by the U.S. National Consortium for Public Health Workforce Development.^16^

The capacity indicators for the pilot tool were chosen based on workforce groups that were listed in WHO JEE and SPAR workforce indicators, as well as the ECDC workforce capacity and training needs survey. The most relevant workforce groups regarding NPHIs were identified through discussions within the working group. As a result, we selected five specific PHEP workforce groups as indicators for which the tool would assess both ‘peace-time’ and surge capacity requirements: (i) public health epidemiologists, (ii) public health microbiologists, (iii) data scientists/statisticians, (iv) modellers and (v) communication experts. Furthermore, Total workforce in PHEP and Total workforce at the NPHI were selected as indicators. As the eighth indicator, we chose the Proportion (%) of total NPHI staff in PHEP, calculated as follows: Proportion (%) of total NPHI staff in PHEP = Total workforce in PHEP/Total workforce at the NPHI.

Competencies were assessed using a modified Hennessy–Hicks TNA.^11^ Here, items (skills, tasks or competencies) are scored on a seven-point scale according to two criteria: ‘How critical the task is to the successful performance of the respondent’s job’ and ‘How well is the task currently performed’. The questionnaire can identify how best to improve performance in areas where deficits have been identified: in addition to importance and performance measures, each item can also be rated along a seven-point scale according to how far the respondent believes that the competency gap can be addressed by organizational changes or training. As we validated the importance of each competency indicator through a Delphi process involving end-users, in the final version of the tool, we omitted the rating of importance. For improving performance, instead of the original two options (organizational changes and training), we selected three alternatives that are not mutually exclusive: (i) training the current PHEP staff performing the function, (ii) training other staff from your organization to perform the function and (iii) recruiting new staff. This selection was supported by the results of focus group discussions, where it was suggested a similar approach for a three-level surge capacity system in a Public Health Emergency Operations Centre (Level 1: work in the usual core group; Level 2: draw resources from the same institute to upscale the staff; Level 3: recruit outside of the organization).

### Review and refinement of the PANDEM-2 workforce self-assessment tool

#### Responses from questionnaire

From the eight quantitative indicators (capacities) in the pilot tool version, the Delphi questionnaire respondents agreed to include seven indicators. One indicator was nominated for discussion during the consensus round. Respondents suggested an additional seven capacity indicators which were discussed and voted on in the consensus round (table 1).

**Table 1:** Summary of the Delphi questionnaire results which demonstrates the initial number of indicators in the pilot tool and how this evolved based on outcomes of the Delphi questionnaire.

Number of indicators	Quantitative indicators	Qualitative indicators	All indicators
Starting number of indicators (pilot tool)	8	115	123
Included	7	98	105
Excluded	0	15	15
Discussed/voted	1	2	3
Additional indicators suggested, to be discussed/voted	7	2	9
Total number of indicators to be discussed/voted	**8**	**4**	**12**

Notes: An in-depth explanation of indicators excluded is available in table 2. In the consensus meeting, a total of 12 indicators (eight quantitative; four qualitative were discussed and voted on). The results of these discussions are outlined in table 3.

Green = included, Red = excluded, Yellow = discussed/voted on in the consensus meeting.

From the 115 qualitative indicators (competencies), responses to the questionnaire resulted in 98 indicators being included in the final tool, and 15 indicators were excluded based on feedback. Two indicators were nominated for discussion with an additional two indicators suggested by respondents. Therefore, four qualitative indicators were included for discussion in the consensus round (table 1).

A summary of the 15 qualitative indicators excluded based on the analysis criteria can be found in table 2. The excluded indicators were mainly from category 3 Healthcare: respondents indicated that these indicators are not the responsibility of the NPHI, but mainly of authorities such as the Ministry of Health or the healthcare sector.

**Table 2:** Indicators excluded based on questionnaire analysis criteria.

Organization-wide competencies		Median	Agreement	Comments
**1. Detection and assessment**				
**1.4 Surveillance and epidemiological monitoring**	Develop and implement plans for border screening for known pathogens of international concern.	7	71%	Important but not done in real time
**2. Policy development, adaptation, and implementation**				
**2.1 Policy development and adaptation for infection control and treatment guidance**	Aid the transfer of medical and related professionals across borders and facilities through standardized job descriptions of personnel in clinical settings.	6	46%	Not performed by NPHI
**2.3 Policy implementation**	Before the response operation, ensure the adequacy of plans for financing and credentialing of staff during emergency situations.	7	69%	Financing is a regional responsibility. Unclear on what credentialling here means
**3. Health Services**				
**3.2 Medical surge**	Prior to an event, work in tandem with clinicians to develop medical surge plans for various threats.	7	54%	Very important, although is not performed by NPHI (alone)
	Ensure that plans across the continuum of care have been communicated to the clinical staff to effectively manage surge needs.	6	46%	Not performed by NPHI (alone)
	Plan for combining resources at national and local levels (e.g. cross-border sharing of clinicians if a hospital reaches capacity).	6	38%	Not performed by NPHI (alone)
	Establish processes for staffing related surge issues including credentialing, paying staff, channels of authority, extended crisis interventions and livelihood protection at home.	6	38%	Not performed by NPHI (alone)
	Create a hospital-based unit for critical, contagious patients at select facilities known to medical evacuation teams.	5.5	42%	Not performed by NPHI (alone)
**3.3 Management of medical countermeasures, supplies and equipment**	Ensure flexible policies and procurement strategies among MSs including how to allocate resources in the event of a shortage.	6	46%	Not performed by NPHI (alone)
	Ensure there are adequate levels of human resources (e.g. experts) and laboratory capacity available in the MSs.	6.5	50%	Not performed by NPHI (alone)
**3.4 Medical services for healthcare workers and emergency responders**	Before a response operation, relay to healthcare workers the importance of their role in public health emergencies and support their personal preparedness and that of their families.	7	54%	Not performed by NPHI (alone)
	Establish ways to procure PPE for medical professionals and emergency responders across MSs.	6	50%	Not performed by NPHI (alone)
	Plan for the demobilization and recovery of the healthcare workforce after a response operation.	5	50%	Not performed by NPHI (alone)
**5. Emergency risk communication (with the public)**				
**5.4 Foster and maintain trust**	Empower the public to participate in open discussions; involve the public in decisions relevant to public health threats.	7	71%	Not performed by NPHI (alone). Important, but benefits to response still unclear.
**6. Cross-cutting competencies**				
**6.4 Resource management**	Manage recruitment and career paths of the workforce as well as acquisition, retention, and management of fiscal resources.	7	77%	Unclear which workforce is meant.

Note: As outlined above, the majority of indicators were excluded as they were activities deemed to be outside the remit of a National Public Health Institute.

The colours in Table 2 are used to differentiate between the different indicators and sub-groups. The colours themselves do not have any relevance.

#### Consensus round

In the consensus round, the three indicators nominated for discussion/voting (table 1) were rejected (see table 3 for information).

**Table 3:** Indicators discussed and voted on the Delphi consensus round including respondent comments and decision/modification made in terms of inclusion in the final PANDEM-2 workforce capacity self-assessment tool.

Indicator type and source	Indicator title	Votes to ‘include’	Respondent comments	Decision and/or modification to final tool
Quantitative, questionnaire	Proportion of total NPHI staff in PHEP	58%	Not a clear indicator. Preparedness is difficult to define.	Excluded.
Quantitative, suggested	Practical coordinators or managers	83%	Important workforce during COVID-19. Suggest combining with other indicators as ‘Practical organization’ or ‘Management and support’.	Included, modified as ‘Management and support’, which partially includes roles in suggested indicators ‘Workforce for public health response’ and ‘Available staff for EOC’.
Quantitative, suggested	Public health doctors	83%	Suggest specifying as ‘Doctors with expertise in Infectious Diseases and Public Health’.	Included, combined with ‘IP specialists’ and modified as ‘Doctors with expertise in Infectious Diseases and Public Health, and other specialists in IPH’.
Quantitative, suggested	Infection prevention specialists	67%	IPH specialists are not always doctors, so a highly relevant indicator. Suggest combining with ‘Public health doctors’.	Included, combined with Public health doctors, modified as ‘Doctors with expertise in Infectious Diseases and Public Health, and other specialists in IPH’.
Quantitative, suggested	Workforce focusing on IHR implementation	58%	Do not see the added value here: IHR implementation is already a core part of standard work in preparedness and response.	Excluded. The implementation of IHR is included in part 2.3 (‘Policy implementation’) of the pilot tool.
Quantitative, suggested	Workforce for public health response (creating guidelines, organization of rapid response teams)	58%	Suggest combining with other indicators.	Excluded. The role is included in the combined indicators ‘Management and support’ and ‘Doctors with expertise in Infectious Diseases and Public Health, and other specialists in IPH’.
Quantitative, suggested	Available staff for EOC	50%	Suggest combining with other indicators.	Excluded. The role is included in the combined indicators ‘Management and support’ and ‘Doctors with expertise in Infectious Diseases and Public Health, and other specialists in IPH’.
Quantitative, suggested	Staff absenteeism due to sickness/personal reasons during pandemic	58%	Context driven. Usefulness unclear. Confounding factors difficult to control.	Excluded.
Qualitative, questionnaire	3.1 Facilitate the approval of vaccines through streamlined processes where available.	8%	Not the responsibility on NPHI.	Excluded.
Qualitative, questionnaire	4.4 Assess the adequacy of mutual aid mechanisms and multidisciplinary taskforces.	25%	The meaning is unclear.	Excluded.
Qualitative, suggested	Collaboration with universities in training NPHI and healthcare staff in response activities and applied research methods	92%	Used in our country to increase the surge capacity for contact tracing. Can also be another training organization (instead of universities). Is this a capability?	Included, modified as ‘Advocate the development of plans to collaborate with universities and other training organizations to meet training needs under a PHE (e.g. contact tracing)’.
Qualitative, suggested	Collaboration with universities in maintaining surge capacity for certain workforce needs (i.e. epidemiology, statistics, modelling etc.)	92%	This was the case with COVID-19 response in our country. Reasonable, but how it’s done depends on context. Relevant, a valuable addition. Overlapping with the previous indicator?	Included, modified as ‘Advocate the development of plans to receive surge workforce capacity from universities to the NPHI (e.g. epidemiologists, statisticians and modellers)’

In response to the questionnaire, participants suggested a total of nine additional (seven quantitative, two qualitative) indicators to be discussed and voted on in the consensus round. Several were rejected in their original form but based on participant feedback, some were modified and partly combined with previously accepted indicators. Consequently, from the initial nine additional indicators suggested, two quantitative and two qualitative indicators were added to the final tool. These include the following:

Quantitative indicators:

1. Workforce in PHEP at NPHI: Management and support.
2. Workforce in PHEP at NPHI: Doctors with expertise in Infectious Diseases and Public Health, and other specialists in infection prevention and hygiene.

Qualitative indicators:

1. Advocate the development of plans to collaborate with universities and other training organizations to meet training needs under a PHE (e.g. contact tracing).
2. Advocate the development of plans to receive surge workforce capacity from universities to the NPHI (e.g. epidemiologists, statisticians and modellers).

A copy of the final PANDEM-2 workforce capacity self-assessment tool^17^ is available on Zenodo (https://zenodo.org/records/8232666).

## Discussion

In collaboration with six EU MS NPHI, we developed and validated a tool that enables the assessment of the PHEP workforce both in quantitative (FTEs) and qualitative (competency) terms. Through a review of existing resources and focus group discussions, we have identified indicators and metrics relevant to the PHEP context. This tool specifically addresses the current gap in the assessment of PHEP workforce needs and requirements of NPHIs at the institutional level. This tool can be used to assess the needed workforce for PHEP functions during normal circumstances, and the availability of surge capacity under a Public Health Emergency situation. Furthermore, the tool enables NPHIs to analyze the gaps in PHEP staff competencies. Subsequently, the assessment scores can be used to identify and prioritize training and recruitment needs.

A major gap identified in this study was the distinct lack of a central registry of public health or PHEP professionals, and that the numbers of staff are not available to the NPHIs. This finding is supported by the Assessment of workforce capacity and training needs for the prevention and control of communicable diseases in the EU/EEA (2022).^18^ The WHO is currently addressing the challenge of workforce enumeration through a roadmap to define, map and measure the workforce delivering essential public health functions with a specific focus on emergency preparedness and response.^19^ Due to the existing challenges and the ongoing larger WHO initiative, the PANDEM-2 workforce self-assessment tool was designed to help address these challenges by providing a way to measure total NPHI staff capacity and emergency preparedness at the institutional level. The tool will also provide a useful benchmark for NPHIs to advocate for additional investment in the PHEP workforce. Acknowledging that the organization of PHEP functions in the European countries differ, the tool may also be used at regional Public Health Agency level, especially in those countries where public health functions are decentralized.

Information from such an assessment will be highly relevant in future, as the EU is currently taking actions to implement activities under the article 11 ‘Training of healthcare staff and public health staff’ of Regulation (EU) 2022/2371 on serious cross-border threats to health.^13^ These actions will include the assessment of current workforce capacity and capability gaps in EU MSs. The PANDEM-2 workforce self-assessment can therefore align with this need and will help effective planning and development to strengthen the workforce capacity in EU MSs.

## Supplementary Material

ckae030_Supplementary_Data

## Data Availability

The data relating to the PANDEM-2 workforce capacity tool outlined in this article are available in Zenodo and can be accessed at https://zenodo.org/records/8232666. Key points European classification of the Public Health workforce is not yet available Current workforce assessment tools to not provide the required granularity for assessment and planning at sub-national level of a public health agency. The PANDEM-2 workforce self-assessment tool will enable European NPHIs to analyze deficits in PHEP capabilities, ensuring effective planning to strengthen the workforce capacity against future public health threats. This work aligns to article 11 ‘Training of healthcare staff and public health staff’ of Regulation (EU) 2022/2371 on serious cross-border threats to health.

## References

[1] International Health Regulations. IHR State Party Self-Assessment Annual Report (SPAR), 2005. Published online 2018. https://iris.who.int/bitstream/handle/10665/43883/9789241580410_eng.pdf?sequence=1 (18 January 2023, date last accessed).

[2] Internal Health Regulations. State parties self-assessment annual reporting tool. The World Health Organisation report no. WHO/WHE/CPI/2018.16, 2005. https://extranet.who.int/sph/sites/default/files/document-library/document/WHO-WHE-CPI-2018.16-eng_1.pdf (5 August 2022, date last accessed).

[3] World Health Organization. Joint External Evaluation Tool: International Health Regulations (2005), 3rd edn., 2022. Geneva: World Health Organization; 2022. ISBN: 9789240051980.

[4] European Centre for Disease Prevention and Control. HEPSA—Health Emergency Preparedness Self-Assessment tool, 2017. Available at: https://www.ecdc.europa.eu/en/publications-data/hepsa-health-emergency-preparedness-self-assessment-tool (14 April 2023, date last accessed).

[5] Myhre SL , French SD, Bergh A. National Public Health Institutes: a scoping review. Glob Public Health 2022;17:1055–72. 33870871 10.1080/17441692.2021.1910966

[6] Frieden TR , Koplan JP. Stronger National Public Health Institutes for global health. Lancet 2010;376:1721–2. 21093637 10.1016/S0140-6736(10)62007-7 PMC7138417

[7] Koplan JP , Puska P, Jousilahti P, et al; National Public Health Institute Partners. Improving the world’s health through National Public Health Institutes. Bull World Health Organ 2005;83:154–7. 15744409 PMC2623816

[8] European Centre for Disease Prevention and Control. Assessment of workforce capacity and training needs for the prevention and control of communicable diseases in the EU/EEA. Report on 2021 survey. Stockholm: ECDC; 2022. 10.2900/453712 (3 August 2022, date last accessed).

[9] Council on Linkages between Academia and Public Health Practice. Core competencies for public health professionals, 2021. Available at: https://www.phf.org/resourcestools/pages/core_public_health_competencies.aspx (14 April 2023, date last accessed).

[10] de Beaumont Foundation. Adapting and aligning public health strategic skills, 2021. Available at: https://debeaumont.org/strategic-skills/ (10 January, 2023, date last accessed).

[11] Markaki A , Malhotra S, Billings R, et al Training needs assessment: tool utilization and global impact. BMC Med Educ 2021;21:310. 34059018 10.1186/s12909-021-02748-y PMC8167940

[12] Fitch K , Bernstein SJ, Aguilar MD, et al The RAND/UCLA Appropriateness Method User's Manual. Santa Monica, CA: RAND Corporation, 2001.

[13] European Parliament, Council of the European Union. Regulation (EU) 2022/2371 of the European Parliament and of the Council of 23 November 2022 on serious cross-border threats to health and repealing Decision No 1082/2013/EU (Text with EEA relevance). http://data.europa.eu/eli/reg/2022/2371/oj (14 April 2023, date last accessed).

[14] European Centre for Disease Prevention and Control. Public health emergency preparedness—core competencies for EU member states, Stockholm: ECDC; 2017. 10.2900/049462 (10 January 2023, date last accessed).

[15] European Centre for Disease Prevention and Control. Core competencies in applied infectious disease epidemiology in Europe, Stockholm: ECDC; 2022. 10.2900/657328 (10 April 2023, date last accessed).

[16] de Beaumont Foundation. Building Skills for a More Strategic Public Health Workforce: A Call to Action U.S. National Consortium for Public Health Workforce Development. Available at: https://publichealthworkforcedevelopment.org/ (10 January 2023, date last accessed).

[17] Voutilainen Liina, Lindh Erika, Barreto Marta, Mexia Ricardo, Hayes Jessica, & Connolly Máire. (2023). PANDEM-2 Workforce capacity tool for public health emergency preparedness. Zenodo. 10.5281/zenodo.8232666 PMC11161143 38561183

[18] European Centre for Disease Prevention and Control. Assessment of workforce capacity and training needs for the prevention and control of communicable diseases in the EU/EEA. Report on 2021 survey, Stockholm: ECDC; 2022. 10.2900/453712 (12 August 2022, date last accessed).

[19] National workforce capacity to implement the essential public health functions including a focus on emergency preparedness and response: roadmap for aligning WHO and partner contributions. Geneva: World Health Organization; 2022.

