# Supplementary material for: Development of a workforce self-assessment tool for public health emergency preparedness
Source: Eur J Public Health. 2024 Apr 1;34(3):482–9. doi: 10.1093/eurpub/ckae030 (PMC11161143; doi:10.1093/eurpub/ckae030)
Supplement: ckae030_Supplementary_Data [file ckae030_supplementary_data.pdf]

Supplementary information

Development of a workforce self-assessment tool for public health emergency preparedness (Hayes *et al.,*2024)

1. Focus group questions

1) What are NPHI's experiences on existing assessment methods by WHO and ECDC (such as JEE, SPAR, HEPSA, ECDC surveys on workforce capacity assessment and training needs)
   a) Please describe your experience with WHO/ECDC tools if any
   b) Usability vs. effort - are the WHO/ECDC tools useful in relation to the effort needed completing them (evaluations, surveys, questionnaires)
      i) What have the main challenges been when completing the tools? For example: were data easy to find, or the right people to provide it? Was the information easy to put on the given scale?
      ii) Are these methods used for personnel/HR planning at your institute (or are they separate from your planning)? Are there plans to fill the identified capacity/capability gaps?
      iii) Are there gaps in the existing tools? Especially looking back to covid-19, are there areas/themes/indicators that should be included in assessment tools?

2) How do NPHI's assess and plan their workforce capacity and capability (best practices)?
   a) Please describe the respondent's role in workforce capacity and capability planning at your institute, if any
   b) Does your organization have a formal plan or any other best practice for PHEP workforce capacity/capability assessment?
      i) If yes, does it take into account scaling up/relocating of functions/personnel?
      ii) Has that plan been updated after the onset of covid-19?
      iii) Does it cover some other element that is not covered by the WHO and ECDC tools?

iv) Is there an inventory of the PHEP workforce in your country?

3) What would an ideal tool for workforce assessment at PHIs be like?

   a) How should an assessment be completed in your organization (meeting/workshop, circulation in email/online)

   b) How often should the assessment be repeated? (For example, 1/3/5 years)

   c) What kind of scaling is appropriate (for example numbers of staff, FTEs per function, % out of needed, ratings (1-5), “traffic light” colours)

   d) Which functions/roles at your organization should be involved in the assessment (HR, leaders/managers, substance experts, financial, other)? What is the ideal number of people to involve?

   e) Should there be themes that are assessed by *each* PHEP workforce staff member?

4) Any other comments and thoughts on workforce capacity and capability assessment in the context of public health emergency preparedness at national public health institutes

2. A review of existing tools for workforce assessment

Source	Summary
WHO States Parties Annual Reporting (SPAR) tool	- 24 indicators for the 13 IHR capacities needed to detect, assess, notify, report, and respond to public health risk and acute events of domestic and international concern<br>- Regarding human resources, SPAR evaluates if strategies are in place to ensure that a multisectoral workforce is available and trained to enable early detection, prevention, preparedness, and response to potential events of international concern at all levels of health systems, as required by the IHR
WHO Joint External Evaluation (JEE) tool	- voluntary, collaborative, multisectoral process to assess country capacities to prevent, detect and rapidly respond to public health risks whether occurring naturally or due to deliberate or accidental event<br>- helps countries identify the most critical gaps within their human and animal health systems to prioritize opportunities for enhanced preparedness and response.<br>- considered to be a good starting point for National Action Plans aimed at improved response capacity and capability<br>- first external evaluation establishes a baseline measurement of the country's capacity and capabilities, and subsequent evaluations identify progress made and sustainability of improvements.<br>- Involves self-evaluation followed by external review<br>- The JEE tool supports the external evaluation process including development of recommendations across 19 technical areas, including human resources.<br>- The JEE can also serve as a mechanism to validate the results of the SPAR.
ECDC Assessment of workforce capacity and training needs for the prevention and control of communicable diseases in the EU/EEA	- Conducted every 3 years<br>- Used by countries to map the size and composition of their existing workforce capacities, and to quantify, qualify and prioritise training needs to enable the planning of ECDC training activities.<br>- survey consists of two parts: a Workforce Capacity Assessment Survey and a Training Needs Assessment Survey.<br>- In each country, the Workforce Capacity Survey is administered through the ECDC National Coordinator, and the Training Needs Survey through the ECDC National Focal Point for Training.<br>- Capacity is assessed through estimating the FTE of staff currently working in CD prevention and control in public health institutions, for the following job functions: Public Health Epidemiologist, Public Health Microbiologist/Genomics Specialist, Public Health Veterinarian, Sociologist/Anthropologist, Sanitarian or Environmental Specialist, Informatics Specialist/Data Manager, Statistician/Mathematical Modeller, Communication specialist, Infection Prevention and Control/Hospital Hygiene Specialist, Capacity Building/Training Specialist, Health Economist and Other Public Health Practitioner (including medical doctor, nurse, midwife, pharmacist).<br>- training needs are assessed through setting a priority score (high, medium or low) for seven core competencies (Public health emergency preparedness, Prevention, Surveillance, Risk assessment, Laboratory system and methods, Communication and advocacy, and Response).
ECDC HEPSA tool	- aims to aid countries in improving their level of PHEP by evaluating levels of preparedness, identify potential gaps, identify vulnerabilities, and detect areas for improvement<br>- consists of seven domains covering all areas of public health preparedness and response

	- HEPSA includes one relevant domain named Resources: trained workforce (domain 2). If necessary, the HEPSA tool allows assessing only this domain instead of all seven domains.<br>- two sets of allocated indicators to measure and monitor the level of preparedness; (1) the set of eight baseline indicators define basic country preparedness, which should be achieved in every country, irrespective of how the country's health system is structured; (2) The set of comprehensive indicators all eight baseline indicators and five additional indicators that provide a more comprehensive view of the domain.<br>- The score calculation can be based on either baseline or comprehensive indicator sets.<br>- In practise, HEPSA is a macro-enabled Microsoft Excel workbook, where each of the indicators is scored on percentage scales for frequency (0% = "Never", 100% = "Always") and achievement (0% = "Not achieved, no progress", 100% = "Comprehensive achievements with sustained commitment and capabilities at all levels").<br>- HEPSA tool contains cross references to the WHO Joint External Evaluation tool which helps users calculate potential JEE scores and assess their preparedness level.
ECDC Public health emergency preparedness: Core competencies for EU Member States	- describes the competencies that different groups of professionals need – in addition to the basic knowledge and skills required for their profession – when they are called upon to serve as members of national preparedness committees or teams in order to facilitate an effective national and cross-border response to health threats in Europe<br>- The competency-based model proposes five public health preparedness capability categories: (1) Detection and assessment, (2) Policy development, adaptation, and implementation, (3) Healthcare services, (4) Coordination and communication (within the public health emergency preparedness system), and (5) Emergency risk communication (with the public).<br>- Each of these five categories includes three to six capabilities, for which respective competencies, i.e., combinations of knowledge and skills required to perform a task effectively, are determined (one to eight competencies per each capability).<br>- for each capability, up to 20 statements of required knowledge and skills are listed.<br>- Combined, the model includes 102 competencies and 258 knowledge and skill statements.
ECDC Core competencies in applied infectious disease epidemiology in Europe	- lists 157 competencies grouped into six subject areas: (1) Essential methods for applied infectious diseases epidemiology, (2) Preparedness, surveillance and response to infectious disease outbreaks, (3) Communication and advocacy, (4) Practice of infectious disease epidemiology, (5) Contextual influences on infectious disease management and (6) Leadership and management<br>- Each subject area is broken down into three or four cross-linked domains that each include 3 to 10 competencies<br>- The framework was launched with a companion toolkit that enables individuals to evaluate their level in each competency, available in the ECDC Virtual Academy EVA<br>- In the process of updating the current competency framework from its previous version, new and emerging areas of expertise were recognised, and consequently, new domains "Infodemiology and infodemic management" and "One Health, environment and climate change" were added.
Other: The Council on Linkages Between Academia and Public Health Practice: Core Competencies for Public Health Professionals	- contain 56 competency statements that apply across the public health workforce for those engaged in the practice, education, and research of public health.<br>- competency statements are organized into eight domains representing skill areas within public health (data analytics and assessment skills, policy development and program planning skills, communication skills, health equity skills, community partnership skills, public health sciences skills, management and finance skills, leadership and systems thinking skills)

	- In addition, the Core Competencies include three tiers, which describe different types of responsibilities within public health organizations (front line and program support responsibilities, program management and supervisory responsibilities and senior management and executive leadership responsibilities).
Other: De Beaumont foundation: Adapting and Aligning Public Health Strategic Skills	- in 2017, the de Beaumont Foundation led the development of the National Consortium for Public Health Workforce Development comprised of public health leaders from 34 national partner organizations representing a variety of disciplines and settings in the US<br>- The Consortium identified the following nine "indispensable, high-performance skills applicable to the entire public health workforce regardless of specialty or discipline.": Systems and strategic thinking, change management, Effective communication, Data-driven decision making, Community engagement, Justice, equity, diversity, and inclusion, Resource management and finance, Policy engagement, and Cross-sectoral partnerships.<br>- In 2021, the de Beaumont Foundation has published a report in which it presents refreshed definitions for the Strategic Skills and a crosswalk of these renewed Strategic Skills to the Core Competencies as a foundational first step toward advancing public health's mission and issue recommendations.

3. Pilot workforce self-assessment tool

Pilot workforce capacity self-assessment tool that was reviewed by participants of 6 national public health agencies. The tool was developed based on pre-existing literature and feedback from focus-groups. The pilot tool was refined using a Delphi study design. The final version of the tool can be found at https://zenodo.org/records/8232666

WORKFORCE CAPACITY	PUBLIC HEALTH INSTITUTE (PHI) REVIEW - DELPHI STUDY		DO NOT FILL IN - FOR DEMONSTRATION OF THE TOOL	
	**Rate the relevance of the indicator regarding the role and mandate of your organisation, looking back to COVID-19 pandemic** (Select from drop-down list)	**Reason why nonrelevant** (Free text)	FTE's* **under normal circumstances**	FTE's available for **surge capacity during a PH emergency**
Total workforce in Public Health Emergency Preparedness at National Public Health Institute				
Workforce in Public Health Emergency Preparedness at National Public Health Institute: **public health epidemiologists**				
Workforce in Public Health Emergency Preparedness at National Public Health Institute: **public health microbiologists**				
Workforce in Public Health Emergency Preparedness at National Public Health Institute: **data scientists/statisticians**				
Workforce in Public Health Emergency Preparedness at National Public Health Institute: **modellers**				
Workforce in Public Health Emergency Preparedness at National Public Health Institute: **communication experts**				
Total workforce at National Public Health Institute				
Proportion of total PHA staff in PHEP			Percentage (%)	Percentage (%)

Please indicate below any other relevant quantitative indicator (workforce group, level of assessment, etc.)

Suggested indicator 1
Suggested indicator 2
Suggested indicator 3
Suggested indicator 4
Suggested indicator 5

*FTE's = full-time equivalents, i.e. an employee's scheduled hours divided by the employer's hours per a time period, equivalent to person weeks/months/years. The time period may be adjusted according to a time span that is relevant for a PH emergency.

PUBLIC HEALTH EMERGENCY PREPAREDNESS CAPABILITIES proposed in *Public health emergency preparedness: Core competencies for EU Member States* (ECDC 2017) https://www.ecdc.europa.eu/en/publications-data/public-health-emergency-preparedness-core-competencies-eu-member-states		ORGANIZATION-WIDE COMPETENCIES	PUBLIC HEALTH INSTITUTE (PHI) REVIEW - DELPHI STUDY		DO NOT FILL IN - FOR DEMONSTRATION OF THE TOOL			
			**Rate the relevance of the competency regarding the role and mandate of your organisation, looking back to COVID-19 pandemic** (Select from drop-down list)	**Reason why nonrelevant** (Free text)	**How well is the function performed?** (overall score for the organization: not well = 1; very well = 7)	Could performance be improved through **training the current PHEP staff** performing the function? (1 = not at all, very much = 7)	Could performance be improved through **training other staff** from your organisation to perform the function? (1 = not at all, very much = 7)	Could performance be improved through **recruitment of new staff**? (1 = not at all, very much = 7)
**1. DETECTION AND ASSESSMENT**								
**1.1 Incident recognition**								
**1.1 Incident recognition**	Identifying that a health threat with cross-border potential has arisen, either in one or more of the Member States, or elsewhere in the world that could affect Europe.	Use event-based and indicator-based surveillance systems to detect health threats.			2	7	5	2
	Workforce groups: Public health epidemiologists, national public health agency leaders, NFP for preparedness	Know when case reports or clusters require further investigation, and how to initiate such investigations.						
		Evaluate the implications of national or international public health alerts for own Member State.						
**1.2 Risk characterisation**	For communicable diseases, identifying the (possibly novel) pathogen and its epidemiologic characteristics such as reservoir and potential sources, modes of transmission, risk groups, level and duration of infectiousness, virulence (e.g. case–fatality rate), generation time, available control strategies. Based on this, assess the risk. For other health risks, characterising the current and potential consequences for human health in directly affected and other Member States.	Identify as rapidly as possible the (possibly novel) agents responsible for a disease outbreak and their epidemiological characteristics.						
		Update estimates of an agent's epidemiologic characteristics as new information becomes available.						
	Workforce groups: Public health epidemiologists, public health microbiologists, national public health agency leaders	Characterise the current and potential human health consequences of population exposure to biological, chemical, radiological and nuclear hazards.						
		Perform a risk assessment.						
		Apply the results of international risk assessments to own Member State.						
		Communicate the results and implications of risk assessments for their own Member States to policymakers with different backgrounds.						
		Communicate the results and implications of risk assessments to those responsible for emergency risk communication.						
**1.3 Epidemiological investigation**	Developing case definitions, conducting outbreak investigations and casecontrol studies to validate and analyse case reports, identify pathogens and sources of exposure, and to aid in risk characterisation.	Develop case definitions to validate and analyse case reports.						
	**Workforce groups:** Public health epidemiologists, public health microbiologists, NFP for preparedness	Conduct outbreak investigations to identify pathogens and other agents, characterise affected population groups, and sources of exposure.						
		Conduct case-control studies and other epidemiologic studies to test hypotheses regarding sources of exposure.						
		Collaborate with local health officials, healthcare providers, and others to conduct outbreak investigations and epidemiologic studies.						
		Collaborate with international organisations to conduct coordinated multinational epidemiologic studies.						

**PUBLIC HEALTH EMERGENCY PREPAREDNESS CAPABILITIES** proposed in *Public health emergency preparedness: Core competencies for EU Member States* (ECDC 2017) https://www.ecdc.europa.eu/en/publications-data/public-health-emergency-preparedness-core-competencies-eu-member-states		**ORGANIZATION-WIDE COMPETENCIES**	**PUBLIC HEALTH INSTITUTE (PHI) REVIEW - DELPHI STUDY**		**DO NOT FILL IN - FOR DEMONSTRATION OF THE TOOL**			
			**Rate the relevance of the competency regarding the role and mandate of your organisation, looking back to COVID-19 pandemic** (Select from drop-down list)	**Reason why nonrelevant** (Free text)	**How well is the function performed?** (overall score for the organization: not well = 1; very well = 7)	Could performance be improved through **training the current PHEP staff** performing the function? (1 = not at all, very much = 7)	Could performance be improved through **training other staff** from your organisation to perform the function? (1 = not at all, very much = 7)	Could performance be improved through **recruitment of new staff**? (1 = not at all, very much = 7)
**1.4 Surveillance and epidemiological monitoring**	Indicator- and event-based surveillance, including case reporting, and active surveillance, to identify outbreaks, characterise affected population groups, monitor disease trends and monitor the impact of control strategies.<br><br>***Workforce groups:*** Public health epidemiologists, public health veterinarians	Establish and maintain indicator and event-based surveillance system(s) to detect public health threats.						
		Establish and maintain electronic real-time reporting systems.						
		Interpret information from existing surveillance in order to characterise affected population groups, and to monitor disease trends and the impact of control strategies.						
		Develop and implement plans for border screening for known pathogens of international concern.						
		Conduct timely and accurate disease reporting in accordance with WHO requirements and consistent coordination with FAO and OIE.						
		Collaborate with local public health officials and the healthcare delivery system, initiate active surveillance to identify additional cases during an epidemiologic investigation.						
**1.5 Laboratory analysis**	Technical ability to identify (possibly novel) pathogens, monitor antimicrobial resistance, and handle large numbers of samples submitted for diagnostic purposes. This capability reflects a Member State's ability to use existing laboratory capacity effectively during an incident in support of the other capabilities listed above under 'Detection and assessment'.<br><br>***Workforce groups:*** Public health microbiologists, national public health agency leaders	Manage a national laboratory system and effective modern point-of-care and laboratory-based diagnostics.						
		Conduct WHO core tests.						
		Participate in multinational epidemiologic studies.						
		Have the biological, clinical, and epidemiological knowledge needed to characterise (potentially novel) pathogens and other agents responsible for an outbreak disease.						
**1.6 Environmental monitoring**	Ability to monitor chemical, biological (including animal), and other contaminants in air, soil, and water.<br><br>***Workforce groups:*** Environmental scientists, national public health agency leaders	Integrate and interpret information from a variety of local, national, and international sources regarding contaminants in air, soil, and water.						

PUBLIC HEALTH EMERGENCY PREPAREDNESS CAPABILITIES<br>proposed in *Public health emergency preparedness: Core competencies for EU Member States* (ECDC 2017) https://www.ecdc.europa.eu/en/publications-data/public-health-emergency-preparedness-core-competencies-eu-member-states		ORGANIZATION-WIDE COMPETENCIES	PUBLIC HEALTH INSTITUTE (PHI) REVIEW - DELPHI STUDY		DO NOT FILL IN - FOR DEMONSTRATION OF THE TOOL			
			**Rate the relevance of the competency regarding the role and mandate of your organisation, looking back to COVID-19 pandemic** (Select from drop-down list)	**Reason why nonrelevant** (Free text)	**How well is the function performed?** (overall score for the organization: not well = 1; very well = 7)	Could performance be improved through **training the current PHEP staff** performing the function? (1 = not at all, very much = 7)	Could performance be improved through **training other staff** from your organisation to perform the function? (1 = not at all, very much = 7)	Could performance be improved through **recruitment of new staff**? (1 = not at all, very much = 7)
**2. POLICY DEVELOPMENT, ADAPTATION, AND IMPLEMENTATION**								
**2.1 Policy development and adaptation for infection control and treatment guidance**	Effective treatment and mitigation of an emergency can begin with clear policy directives and informed policymaking. However, policies must be flexible and adaptable to accommodate for an evolving infection and/or emergency.<br><br>***Workforce groups:*** NFP for preparedness, healthcare infection control experts, public health epidemiologists, public health microbiologists, health officials at the ministry level, public health legal advisors.	Work with epidemiologists, microbiologists, environmental sciences and others to continuously evaluate evidence on patient treatment and infection control.						
		Regularly assess and, as needed, clarify existing policies and/or recommend/advocate measures and communicate them to health officials at the ministry level, border control officials, and others.						
		Share relevant information with healthcare, infection control, and patient transport experts, and solicit their feedback.						
		Seek and receive advice from public health professionals in making border control decisions and reflect to the public how and why these decisions have been made.						
		Be able to use data products from epidemiologists in providing advice in the development of trade and travel restrictions as tools of population-based disease control.						
		Communicate the necessity of policies calling for personal protective measures to mitigate personal risks for the public health professionals.						
		Aid the transfer of medical and related professionals across borders and facilities through standardised job descriptions of personnel in clinical settings.						
		Provide healthcare workers with clinical guidelines for emerging infections from abroad, especially those that may be carried by travellers and the severely contagious.						
**2.2 Policy development and adaptation for population-based disease control**	Authority and practical ability to adapt existing policies and guidance (or develop new if necessary) to prevent spread of communicable diseases; this covers topics as diverse as personal hygiene, social distancing, and border controls.<br><br>***Workforce groups:*** National focal points for preparedness and response, health officials at the ministry level, public health legal advisors, public health emergency response managers, public health epidemiologists	Before the response operation, ensure regular assessments of legal frameworks and propose/advocate measures to address gaps.						
		Before the response operation, assess if the implementation of strategies, plans, and action plans requires any changes in these plans and strategies.						
		Before the response operation, identify which triggers will require key decisions during outbreak response (keeping in mind that triggers may need to be modified to fit specific situations).						
		Review the evidence on current or impending outbreaks; propose and advocate adaptations to policies as needed.						
**2.3 Policy implementation**	Communicating between national and subnational authorities and enforcing laws and regulations. Ability to enforce laws and regulations required for disease control and prevention including the IHR, EU regulations, and Member States' laws and regulations.<br><br>***Workforce groups:*** Health officials at the ministry level, public health epidemiologists, environmental scientists, public health emergency response managers	Communicate policy/guidelines, weigh benefits and costs, understand concerns about implementation, and adapt policies related to border control.						
		Continuously evaluate evidence on threats; communicate if border control policies need to be adapted.						
		Share information with response managers and health officials at the ministry level to support decisions about appropriate countermeasures.						
		Before response activities are taken, regularly review, test, and update the standard operating procedures and ensure that a multi-unit task force is available for the coordination and integration of relevant sectors during response operations.						
		Before the response operation, ensure the adequacy of plans for financing and credentialing of staff during emergency situations.						

PUBLIC HEALTH EMERGENCY PREPAREDNESS CAPABILITIES proposed in *Public health emergency preparedness: Core competencies for EU Member States* (ECDC 2017) https://www.ecdc.europa.eu/en/publications-data/public-health-emergency-preparedness-core-competencies-eu-member-states		ORGANIZATION-WIDE COMPETENCIES	PUBLIC HEALTH INSTITUTE (PHI) REVIEW - DELPHI STUDY		DO NOT FILL IN - FOR DEMONSTRATION OF THE TOOL			
			**Rate the relevance of the competency regarding the role and mandate of your organisation, looking back to COVID-19 pandemic** (Select from drop-down list)	**Reason why nonrelevant** (Free text)	**How well is the function performed?** (overall score for the organization: not well = 1; very well = 7)	Could performance be improved through **training the current PHEP staff** performing the function? (1 = not at all, very much = 7)	Could performance be improved through **training other staff** from your organisation to perform the function? (1 = not at all, very much = 7)	Could performance be improved through **recruitment of new staff**? (1 = not at all, very much = 7)
**3. HEALTH SERVICES**								
**3.1 Preventive services**	Ability of Member States to mitigate a potential event and pre-empt the potential spread of disease through strategies including vaccination, personal protective actions, border measures, and medication distribution.<br><br>***Workforce groups:*** NFP for preparedness, liaison with local public health institutions, public health emergency response managers, health officials at the ministry level, regulatory agency leadership, public-sector healthcare delivery system managers, liaison with non-governmental hospitals and health professionals.	Before an event, plan for the storage and stockpiling of vaccines and prepare for medical and non-medical countermeasures.						
		Draw upon the work of surveillance networks to identify potential events that may indicate the need for the implementation of preventative services plans.						
		Ensure that plans are in place for mass vaccinations and mass prophylactic medication distribution.						
		Coordinate vaccination plans and criteria for vaccination target groups in the Member States to ensure consistency of practices.						
		Facilitate the approval of vaccines through streamlined processes where available.						
		Address antimicrobial stewardship activities.						
**3.2 Medical surge**	Ability to provide adequate medical evaluation and care during events that exceed the limits of the normal medical infrastructure of an affected area during an outbreak of an infectious disease of high impact (IDHI) or other public health incident.<br><br>***Workforce groups:*** NFP for preparedness, liaison with local public health institutions, public health emergency response managers, health officials at the ministry level, regulatory agency leadership, public-sector healthcare delivery system managers, liaison with non-governmental hospitals and health professionals, healthcare infection control experts	Prior to an event, work in tandem with clinicians to develop medical surge plans for various threats.						
		Ensure that plans across the continuum of care have been communicated to the clinical staff to effectively manage surge needs.						
		Plan for combining resources at national and local levels (e.g. cross-border sharing of clinicians if a hospital reaches capacity).						
		Establish processes for staffing related surge issues including credentialing, paying staff, channels of authority, extended crisis interventions, and livelihood protection at home.						
		Establish reliable systems for disseminating case definitions to standardise both the diagnosis and the reporting of case numbers (e.g. confirmed, suspected, probable, or possible cases).						
		Assess laboratory capacity on an ongoing basis and train public health scientists in rapid testing procedures to ensure adequate surge capacity.						
		Create a hospital-based unit for critical, contagious patients at select facilities known to medical evacuation teams.						
**3.3 Management of medical countermeasures, supplies and equipment**	Ability to procure, distribute, and manage countermeasures, supplies and equipment, including personal protective equipment (PPE), during an incident.<br>***Workforce groups:*** NFP for preparedness, public health emergency response managers, health officials at the ministry level, regulatory agency leadership, officials responsible for procurement, and management members of medical products and technology	Work with health personnel to identify the best medical countermeasures based on risk and threat; relay the results of these conversations.						
		Ensure flexible policies and procurement strategies among Member States including how to allocate resources in the event of a shortage.						
		Ensure there are adequate levels of human resources (e.g. experts) and laboratory capacity available in the Member States.						
**3.4 Medical services for healthcare workers and emergency responders**	Ability to provide preventive and medical services to address the physical and mental health needs of healthcare workers and emergency responders.<br>***Workforce groups:*** NFP for preparedness, public health emergency response managers, health officials at the ministry level, public-sector healthcare delivery system managers, liaison workers for non-governmental hospitals, and health professionals	Use standardised approaches across Member States to engage with all personnel who may serve in field operations on the use of PPE.						
		Before a response operation, relay to healthcare workers the importance of their role in public health emergencies and support their personal preparedness and that of their families.						
		Establish ways to procure PPE for medical professionals and emergency responders across Member States.						
		Plan for the demobilisation and recovery of the healthcare workforce after a response operation.						

PUBLIC HEALTH EMERGENCY PREPAREDNESS CAPABILITIES proposed in *Public health emergency preparedness: Core competencies for EU Member States* (ECDC 2017) https://www.ecdc.europa.eu/en/publications-data/public-health-emergency-preparedness-core-competencies-eu-member-states		ORGANIZATION-WIDE COMPETENCIES	PUBLIC HEALTH INSTITUTE (PHI) REVIEW - DELPHI STUDY		DO NOT FILL IN - FOR DEMONSTRATION OF THE TOOL			
			**Rate the relevance of the competency regarding the role and mandate of your organisation, looking back to COVID-19 pandemic** (Select from drop-down list)	**Reason why nonrelevant** (Free text)	**How well is the function performed?** (overall score for the organization: not well = 1; very well = 7)	Could performance be improved through **training the current PHEP staff** performing the function? (1 = not at all, very much = 7)	Could performance be improved through **training other staff** from your organisation to perform the function? (1 = not at all, very much = 7)	Could performance be improved through **recruitment of new staff**? (1 = not at all, very much = 7)
**4. COORDINATION AND COMMUNICATION (WITHIN THE PUBLIC HEALTH EMERGENCY PREPAREDNESS SYSTEM)**								
**4.1 Crisis management**	Employing a systematic approach to organise and manage resources and responsibilities for addressing all aspects of emergencies, including continuity of operations, reporting and evaluation.<br><br>***Workforce groups:*** NFP for preparedness, public health emergency response managers, public health emergency preparedness planners	Continuously create and update an incident management plan that adapts existing policies to the situation at hand.						
		Continuously inform public health emergency response managers about the threat so that the incident management plan can be updated.						
		During the response operation, anticipate resource needs and communicate them to relevant decision makers.						
		Before the response operation, practice and test the ability to make decisions under uncertainty.						
		Participate in the implementation of plans which ensure the continuity of operations.						
		Communicate with political decision makers to mobilise needed resources, communicate current knowledge and uncertainties, and solicit guidance.						
		Before the response operation, identify key assumptions behind plans, identify untenable assumptions, and advocate changes as needed.						
		Develop protocols and test/exercise processes for health emergency operations and their activation.						
**4.2 Communication with healthcare providers**	Communication between public health institutions and healthcare providers, especially regarding surveillance protocols, prevention and treatment guidance, and other matters to ensure coordination of prevention and treatment efforts.<br><br>***Workforce groups:*** NFP for preparedness, public-sector healthcare delivery system managers, liaison with nongovernmental hospitals and health professionals	Before the response operation, establish rapid communication channels within national disease surveillance and healthcare professionals.						
		Before the response operation, establish trust with healthcare providers through feedback loops and two-way communication.						
		For incident communication, draw on clinical personnel trained in risk communication or people involved in the incident, such as doctors or other clinicians.						
		Provide training; include healthcare providers in drills and exercises to test communication lines and avoid communication problems.						
**4.3 Communication with emergency management, public safety, and other sectors**	Communication between public health and other sectors to ensure coordination of prevention and treatment efforts.<br><br>***Workforce groups:*** NFP for public health preparedness, health officials at the ministry level, national public health agency leaders, public health emergency response managers, public health emergency preparedness planners, civil protection agency leadership	Before the response operation, ensure that key partners are familiar with applicable laws, key roles, resources, information needs, and planning assumptions.						
		Before the response operation, ensure adequate preparations for implementing health screening at borders; also ensure that response measures to a public health emergency can be taken right at the point of entry.						
		Advocate the development of plans for joint task forces or other entities which can share information across disciplines.						
		Advocate regular multidiscipline exercises to improve communication with staff and partners.						
		Before the response operation, review mutual aid agreements (where relevant), identify gaps, and propose/advocate solutions to address gaps.						
		Before the response, train staff members in confidentiality policies, chains of evidence, and security issues relating to the exchange of information between partner organisations.						
**4.4 Communication with other public health institutions at the global, European, national, and subnational levels**	Communication between public health institutions at all levels to ensure coordination of prevention and treatment efforts.<br>***Workforce groups:*** NFP for preparedness; health officials at the ministry level, national public health agency leaders	Identify key partners and develop a common understanding of roles, resources, planning assumptions, risks/vulnerabilities, and information that should be shared during response operations.						
		Develop strategies to communicate with professionals who have different skills and knowledge levels; develop strategies to communicate with partner organisations to ensure a coordinated response.						
		Advocate regular multi-country exercises to improve the ability to communicate with partners.						
		Assess the quality of the microbiology networks.						
		Assess the adequacy of mutual aid mechanisms and multidisciplinary taskforces.						

PUBLIC HEALTH EMERGENCY PREPAREDNESS CAPABILITIES proposed in *Public health emergency preparedness: Core competencies for EU Member States* (ECDC 2017) https://www.ecdc.europa.eu/en/publications-data/public-health-emergency-preparedness-core-competencies-eu-member-states		ORGANIZATION-WIDE COMPETENCIES	PUBLIC HEALTH INSTITUTE (PHI) REVIEW - DELPHI STUDY		DO NOT FILL IN - FOR DEMONSTRATION OF THE TOOL			
			**Rate the relevance of the competency regarding the role and mandate of your organisation, looking back to COVID-19 pandemic** (Select from drop-down list)	**Reason why nonrelevant** (Free text)	**How well is the function performed?** (overall score for the organization: not well = 1; very well = 7)	Could performance be improved through **training the current PHEP staff** performing the function? (1 = not at all, very much = 7)	Could performance be improved through **training other staff** from your organisation to perform the function? (1 = not at all, very much = 7)	Could performance be improved through **recruitment of new staff**? (1 = not at all, very much = 7)
**5. EMERGENCY RISK COMMUNICATION (WITH THE PUBLIC)**								
**5.1 Address communication inequalities**	Ability to address differences across population groups on how the message is received, processed, and acted upon due to the socio-economic and cultural characteristics of the population affected by the emergency.<br><br>**Workforce groups:** Health officials at the ministry level, national public health agency leaders, NFP for communication, risk communicators, civil society leadership	Address cultural and societal barriers in the cognitive processing and compliance with recommended behaviours.						
		Use most appropriate content and trusted channels of communication across population groups.						
		Identify strategies to overcome linguistic barriers, e.g. request local assistance.						
**5.2 Use dynamic listening and manage rumours**	Ability to disseminate messages that are clear and collaborate with other organisations, including health professionals and local leaders to disseminate the message through appropriate channels and messengers.<br><br>***Workforce groups:*** Leaders of national public health institutions, NFP for communication, risk communicators, professional journalists, civil society leadership	Identify data gathering mechanisms to understand and monitor the informational needs of the population.						
		Prevent and counter misinformation.						
		Proactively address the needs of the news media and the general public.						
**5.3 Communicate risk in an accurate, transparent and timely manner**	Ability to provide information to the public in a timely manner, taking into account the actual risk and the general public's perception of the risk.<br><br>**Workforce groups:** Ministerial-level health officials, national public health agency leaders, public health epidemiologists, public health microbiologists, NFP for communication, risk communicators, professional journalists, civil society leadership	Integrate the results of the risk-assessment process in the messages						
		Manage and assess situational information received by the organisation						
		Anticipate questions from the public and develop appropriate answers						
		Understand and implement the principles of risk communication						
		Identify strategies to facilitate the release of information (i.e. review outgoing messages in a timely manner)						
		Understand laws and regulations related to ERC						
**5.4 Foster and maintain trust**	Ability to deliver messages that foster citizens' trust in how the government handles an emergency.<br><br>**Workforce groups:** Health officials at the ministry level, national public health agency leaders, NFP for communication, risk communicators, civil society leadership	Provide information to the public on the roles and responsibilities of the various organisations involved in the response operation; try to understand the public's perception of the emergency.						
		Identify strategies to engage with government leaders in order to integrate government priorities and community interests; address concerns that surface during the emergency response.						
		Identify communication mechanisms that are trusted by the public, partners, and community influencers.						
		Empower the public to participate in open discussions; involve the public in decisions relevant to public health threats.						
**5.5 Infodemiology and infodemic management**	*Adopted from ECDC: Core competencies in applied infectious disease epidemiology in Europe (2022)*	Collaborate with specialists in infodemiology to provide credibility to the dissemination of public health information on social media platforms.						
		Promote both the use of evidence-based and evidence-informed decision making for successful infodemic management.						
		In collaboration with communication experts and data scientists, work to ascertain the origin and spread of misinformation on social media platforms.						
		Identify misinformation patterns on different platforms which may increase the risk of infection for certain areas, populations and settings (e.g. testing hesitancy, vaccine hesitancy, resistance to public health advice during an outbreak).						
		Measure and quantify the penetration of infodemics within a population and evaluate approaches for infodemic interventions.						

PUBLIC HEALTH EMERGENCY PREPAREDNESS CAPABILITIES<br>proposed in *Public health emergency preparedness: Core competencies for EU Member States* (ECDC 2017) https://www.ecdc.europa.eu/en/publications-data/public-health-emergency-preparedness-core-competencies-eu-member-states		ORGANIZATION-WIDE COMPETENCIES	PUBLIC HEALTH INSTITUTE (PHI) REVIEW - DELPHI STUDY		DO NOT FILL IN - FOR DEMONSTRATION OF THE TOOL			
			**Rate the relevance of the competency regarding the role and mandate of your organisation, looking back to COVID-19 pandemic** (Select from drop-down list)	**Reason why nonrelevant** (Free text)	**How well is the function performed?** (overall score for the organization: not well = 1; very well = 7)	Could performance be improved through **training the current PHEP staff** performing the function? (1 = not at all, very much = 7)	Could performance be improved through **training other staff** from your organisation to perform the function? (1 = not at all, very much = 7)	Could performance be improved through **recruitment of new staff**? (1 = not at all, very much = 7)
**6. CROSS-CUTTING COMPETENCES adopted from The Council on Linkages Between Academia and Public Health Practice: Core Competencies for Public Health Professionals (2014) and from Beaumont foundation: Adapting and Aligning Public Health Strategic Skills (2021)**								
**6.1. Scientific skills**		Synthesize evidence (e.g., research findings, case reports, community surveys) from print and electronic sources to support decision making						
		Contribute to the public health evidence base (e.g., participating in Public Health Practice-Based Research Networks, community-based participatory research, and academic health departments; authoring articles; reviewing manuscripts; making data available to researchers)						
**6.2 Systems thinking**		Observe patterns and relationships to understand the systems contributing to public health problems and identifying high-impact intervention options.						
**6.3 Problem solving**		Determine the nature of a problem, identify potential solutions, implement an effective solution, and monitor and evaluate results.						
**6.4 Resource management**		Manage recruitment and career paths of the workforce as well as acquisition, retention, and management of fiscal resources.						
		Use performance management systems (e.g., achieving performance objectives and targets, increasing efficiency, refining processes) for organizational improvement.						
**6.5 Change management**		Scale functions up and down or change them entirely in response to the environment.						
		Identify core elements to help sustain functions in challenging times.						
